# Genetic and Environmental Influences on Anxiety Disorders: A Systematic Review of Their Onset and Development

**DOI:** 10.7759/cureus.80157

**Published:** 2025-03-06

**Authors:** Kayleigh A Fox-Gaffney, Pankaj K Singh

**Affiliations:** 1 Medicine, Surrey and Sussex NHS Healthcare Trust, Redhill, GBR; 2 Geriatrics, Surrey and Sussex NHS Healthcare Trust, Redhill, GBR

**Keywords:** disease genetics, environmental science, general anxiety disorder, human genetics and epigenetics, ocd/ anxiety disorders

## Abstract

Fear is an emotion most humans feel throughout their lifetime, often without knowing its exact cause. Fear is considered a behavioural act to escape a potentially threatening situation, whereas anxiety is distinguished by the lack of actual stimuli and, more so, the threat of potential stimuli. Fear and anxiety are two distinct emotions which warrant separate classifications. Understanding both the genetic and environmental influences which contribute to anxiety disorder onset and development can aid in prevention, diagnosis and management; it may also play a role in helping patients further understand their diagnosis and guide future research.

This review examines genetic and environmental contributions to the onset and development of anxiety disorders and explores their implications for treatments and further research.

An extensive search of databases, including PubMed, Web of Science and Google Scholar, using specific search terms led to the collection of a large number of studies prior to further screening. The inclusion criteria were: studies written in English, full-text available, human studies, and studies conducted within the last 10 years (at the time of writing). The exclusion criteria were: animal studies, studies with a focus on neurological anatomy rather than anxiety disorders, and studies including depressive or other psychological disorders. Using a cross-sectional approach allowed for the strengths to be summarised whilst considering the limitations of the research. The studies were screened for limitations and some of these were stated within the research, whilst others had to be interpreted using a subset of pre-formulated questions to ensure reproducibility. Variables such as the main outcomes, conclusions and limitations were tabulated to guide the interpretation of these studies.

Genetic predispositions were linked to specific gene polymorphisms or familial abnormalities in neurological anatomy and often correlated with the likelihood of the onset of anxiety disorders or contributed to the severity of symptoms. Environmental influences were found to affect the functioning of the brain and some studies established the impacts that therapies have on brain function. The majority of studies have implicated that a combination of genetics and environment have an effect on anxiety disorders, with one study suggesting that a single traumatic event can lead to alterations in the function of specific genes related to anxiety disorders.

Both genetic and environmental factors contribute to the onset, development and severity of anxiety disorders, with environmental triggers often influencing the phenotypic expression of these disorders. Further research would benefit from determining specific processes which lead to the onset of anxiety disorders to facilitate their detection and intervention before resulting in life-long and generational consequences. Studies including larger sample sizes and varied subjects would be advantageous in the future.

## Introduction and background

Prevalence of anxiety disorders

Anxiety disorders consist of panic disorder (PD), agoraphobia (AG), social phobia (SP), specific phobias (SPP), post-traumatic stress disorder (PTSD), acute stress disorder, obsessive-compulsive disorder (OCD) and generalized anxiety disorder (GAD). This set of disorders makes up over three-quarters of psychiatric disorders and can account for decreased productivity, greater substance abuse and increased morbidity and mortality [1].

**Figure 1:**
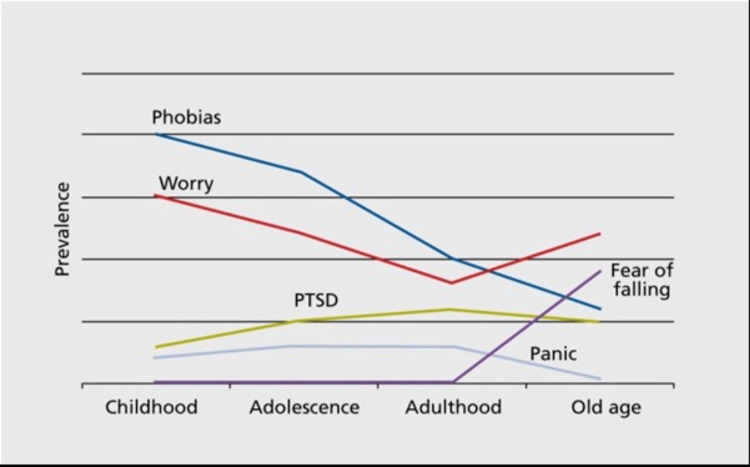
Changes in the prevalence and presentation of anxiety disorders across lifespans Source: Lenze and Wetherell (2011) [4] Figure 1 replicated from Lenze and Wetherell (2011) "A Lifespan of Anxiety Disorders" published in Dialogues in Clinical Neuroscience. (https://pubmed.ncbi.nlm.nih.gov/22275845/) This is an open-access article distributed under the terms of the Creative Commons Attribution License (http://creativecommons.org/licenses/by-nc-nd/3.0/), which permits unrestricted use, distribution, and reproduction in any medium, provided the original work is properly cited.

The lifetime prevalence for anxiety disorders is currently 28%; healthcare providers need to be aware of this prevalence to aid in their management and treatment [2]. One-third of the population suffers from an anxiety disorder and although anxiety disorders can begin at any age, the prevalence generally peaks in middle age and then decreases as individuals get older [3]. Throughout an individual’s lifespan, neurodevelopmental changes occur from childhood to old age (Figure 1). These changes have implications on the cause, presentation, management and treatment of anxiety disorders and, due to the deleterious effects it can have, it is important to understand the disorder and the risk factors that may contribute to its onset [4].

Risk factors for anxiety disorders and environmental influences

Sex

Females are twice as likely as men to suffer from an anxiety disorder. The cause of this could be a genetic or neurobiological difference in females (Table 1) [3].

**Table 1:** Female-to-male ratio of the prevalence rates of anxiety disorders Source: Bandelow and Michaelis (2015) [3] Table 1 reproduced from Bandelow and Michaelis (2015) "Epidemiology of anxiety disorders in the 21st century" published in Dialogues in Clinical Neuroscience (https://pmc.ncbi.nlm.nih.gov/articles/PMC4610617/). This is an open-access article distributed under the terms of the Creative Commons Attribution License (http://creativecommons.org/licenses/by-nc-nd/3.0/), which permits unrestricted use, distribution, and reproduction in any medium, provided the original work is properly cited. Abbreviations PD: panic disorder; GAD: generalised anxiety disorder; AG: agoraphobia; SAD: social anxiety disorder; SP: specific phobias *Epidemiological surveys: Epidemiologic Catchment Area Program (ECA) (Reiger, Narrow and Rae, 1990) [5]; National Comorbidity Survey-Replication (NCS-R) (Kessler, Petukhova, Sampson et al., 2012) [6]; European Study of the Epidemiology of Mental Disorders (ESEMeD) (Alonso, Angermever and Bernet et al., 2004) [7]; Wittchen et al. - Size and burden of mental disorders in Europe — a critical review and appraisal of 27 studies (Wittchen and Jacobi, 2005) [8]

Study	ECA*	NCS-R*	ESEMeD*		Wittchen et al.
Prevalence rate	1 month	Lifetime	12 months	Lifetime	12 months
PD	2.3	2.1	1.7	1.6	1.8
GAD		1.7	2.6	1.8	2.1
AG		1.6	3.0	1.8	3.1
SAD		1.2	1.6	1.5	2.1
SP	2.2	1.8	2.6	2.1	2.4
All anxiety disorders	2.1	1.5	2.3	1.8	2.1

Education

Individuals with poorer education have a greater prevalence of anxiety disorders [9].

Finance

Those who live or grow up in a low-income household are more likely to suffer an anxiety disorder [9].

Parenting Style

Increased levels of coldness in parents are associated with an increased risk of developing any psychological disorder. Specific to anxiety disorders, parental overprotection and rejection were associated with increased rates of social phobia in offspring [10].

Childhood Adversities

Adverse experiences in childhood such as loss of parents, parental divorce and abuse all increase the risk of anxiety disorders. However, with regard to abuse, it was noted that the onset of anxiety did not begin until it was reported by the individual [9].

Life Events

Different experiences have been shown to result in different psychological disorders. The experience of loss can lead to the onset of depression, whilst the experience of threat events can lead to the onset of anxiety disorders [9].

Genetic factors in anxiety

Family aggregation of anxiety disorders have been found to play a substantial role in the onset and development of anxiety disorders in offspring [11]. Animal studies of mammals have found multiple genes contribute to the susceptibility of developing an anxiety disorder, such as 5-HT1A, 5-HTT, MAO-A, COMT, and CCK-B, amongst others, and the interaction of these genes with the environment helps to determine the overall disease risk [12]. Due to basic neuronal mechanisms being shared across mammalian species, the above genes may also contribute to disease risk in humans and other mammalian species [13].

Impact of anxiety disorders on an individual

Individuals suffering from an anxiety disorder experience a significant impact on their day-to-day life. Anxiety disorders not only have an impact on the individual but can lead to a family burden such as effects on health, psychological well-being and family functioning. A study has shown that this burden should be considered by healthcare professionals to assess the impact on the family and provide management and intervention to reduce burdens and improve the overall quality of life (QOL) for families [14].

Quality of life takes into account the individual's perception rather than assumptions by society or healthcare professionals. A study investigating QOL, using a semi-structured diagnostic interview for individuals with GAD, SP and PD, showed that those suffering with a disorder reported significantly poorer QOL in comparison to non-anxious individuals in the general population. The study also showed that 65% suffered with more than one psychological disorder leading to greater impact on QOL [15].

Anxiety disorders affect financial and occupational functioning. Studies have found that work-loss days in those suffering from an anxiety disorder are higher than somatic disorders such as diabetes [3]. The total healthcare costs to the NHS for anxiety disorders is estimated to be around £8.9 billion [16].

More than 70% of individuals suffering with an anxiety disorder receive no treatment. This is multi-factorial, including lack of knowledge, ignorance to accept, prejudice and discrimination [17]. It is crucial that patients understand the importance of receiving treatment from healthcare professionals; anxiety disorders can lead to a number of debilitating effects, such as suicide risk and greater risk of cardiovascular morbidity and mortality, particularly in women [18].

Fear circuitry and anatomy

Psychological disorders are characterized by several neuroendocrine, neurotransmitter and neuroanatomical abnormalities. Symptoms of anxiety disorders are thought to be a result of disruptions in the emotional centres of the brain. Physiological symptoms of anxiety generally include tachycardia, changes in blood pressure and respiratory distress [19].

Key components of fear circuitry include the amygdala, nucleus accumbens, hippocampus, ventro-medial hypothalamus, periaqueductal grey, thalamic nuclei, insular cortex and some prefrontal regions. The study of animals has allowed the roles of some of these regions to be determined, such as the acquisition of fear conditioning and expression of fear responses in the amygdala, contextual processing in the hippocampus and extinction recall in the infralimbic cortex [20]. Using animal models and functional magnetic resonance imaging (fMRI) has allowed a deeper understanding of the components of the brain that are involved in the fear response. Observations of emotional behaviours have been studied, by the likes of Darwin, and demonstrated similar behaviours across species [21].

Neurotransmitters and neuroendocrine signalling are influential in conducting the fear response. Amino acid neurotransmitters are believed to be involved in the over-activity of the limbic system. Several anxiety disorders have been shown to have dysregulation of the gamma-aminobutyric acid (GABA) inhibitory neurotransmission. Monoamines such as serotonin transporter (SERT) have been studied and evidence has been provided to suggest that lack of SERT can increase the risk of anxiety disorders. Neuropeptides have been shown to have anxiolytic effects in laboratory animals. These effects could be due to interactions between neuropeptides in the limbic regions exerting effects on the amygdala and leading to activation of the behavioural stress response [18].

Although fMRI has been useful in providing some information, clear evidence has not yet been found of a specific fear circuit from fMRI in humans [22].

The amygdala

The amygdala is made up of 13 different subnuclei. The central nuclei (CeA) is the primary output region, the basal nuclei (BA) and lateral nuclei (LA) are used in learning or associative processing in the amygdala; the LA receives information from auditory and visual areas, whilst the BA receives stimuli pathways and process further information from the LA to CeA [23]. It is these subnuclei which generate the physiological response of fear via different components of the brain. [19]

The central nucleus of the amygdala controls the expression of behavioural, autocrine and endocrine responses through different sets of outputs due to a stimulus. A number of cognitive processes contribute to the stimulation of the amygdala to generate the responses to fear. The sensory thalamus can trigger the amygdala through low-level stimuli, whilst the sensory cortex uses more complex processing through the recognition of objects or events, to activate the amygdala. The processing of external stimuli reaches the amygdala via two separate pathways. The direct pathway allows a faster response to potentially dangerous stimuli as it bypasses the sensory cortex; however, this can cause a response to stimuli before there is full awareness of it. The alternative pathway, via the sensory cortex, is able to override the direct pathway in order to rationalise the response to stimuli and determine whether the stimuli require a fear response [24].

Memory formation

Memory is a reconstructive process influenced by thoughts, feelings and behaviours. Learning contributes to the onset of anxiety disorders and, consequently, memory formation [25]. The amygdala plays a pivotal role in the process of memory formation. The basolateral amygdala (BLA) facilitates the influence of stress on the consolidation of memories and often turns them into a source of anxiety [26]. The formation of memories is crucial for survival, as it allows detection and anticipation to avoid potentially dangerous stimuli in future encounters [27].

The acquisition of memories occurs at the time of the experience, likely due to processes which connect a neutral stimulus with the aversive outcome [28]. Studies have shown that adults who have experienced victimization as a child have a significant association with chronic fears, phobias and anxieties [29,30].

The age at which these traumatic events occur could determine whether or not they have a long-term effect. In childhood and adolescence, there is a maturation of the neural circuit which controls fear inhibition, this could suggest that any memories formed during this time could inadvertently lead to an increased fear of that same stimulus into adulthood [31]. Androgens during puberty contribute to the formation of anxiety and fear memory formation. This could suggest that post-adolescence is a crucial time point for changes in anxiety and fear [32].

Rationale for review 

This review aims to synthesize the current research on genetic and environmental influences on anxiety disorders. Current research uses small sample sizes and defined inclusion criteria, making the data difficult to apply to a wider population. Review of the literature will allow the main outcomes and limitations to be consolidated and gaps in individual research identified to aid in future treatment and research directions.

## Review

Methodology

The main search strategy involved extensive searching on databases including PubMed, Web of Science and Google Scholar. The collection of studies used in this review has been narrowed down using specific search terms and use of inclusion and exclusion criteria as below.

Inclusion Criteria

Written in English, full-text available, human studies, conducted within the last 10 years (at the time of writing).

Exclusion Criteria

Animal studies, studies with a focus on neurological anatomy rather than anxiety disorder, and studies including depressive or other psychological disorders.

**Figure 2:**
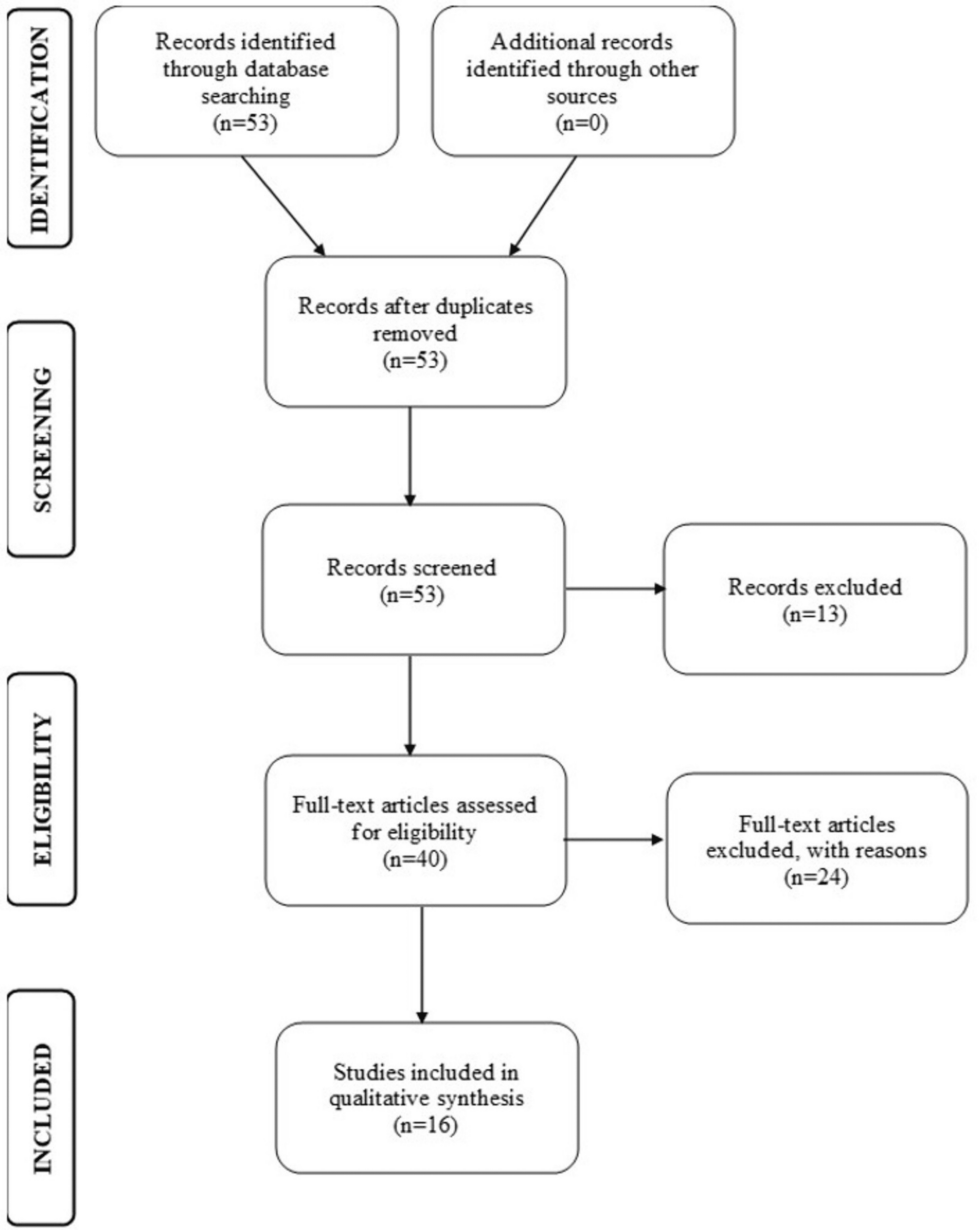
PRISMA flow diagram representing data selection PRISMA: Preferred Reporting Items for Systematic Reviews and Meta-Analyses

The Preferred Reporting Items for Systematic Reviews and Meta-Analyses (PRISMA) flow diagram allows for the process of elimination using screening and the inclusion and exclusion criteria (Figure 2).

The selected research was screened to decipher the number of limitations and any points of bias that could be determined from the study. Some of the limitations were stated within the study by the author, whilst other limitations had to be interpreted using specific questions and guidance. The interpreted limitations considered whether the subject groups had been randomly allocated (where appropriate, whether the subject groups were specific and how the data had been collected, i.e. self-report questionnaires.

Variables such as main outcomes, overall conclusions and limitations were tabulated and used to guide the interpretation of these studies (Table 2). Studies involving pharmacological treatment were assumed to affect physiological functioning and, therefore, imply a genetic factor contributing to the anxiety disorder. Studies involving psychological therapies, such as cognitive behavioural therapy (CBT), were assumed to change the learning and behavioural aspects of anxiety disorders and, therefore, imply an environmental factor. 

**Table 2:** Summary of results from collection of data, outlining the author, year, title, main outcomes and limitations of the studies DTI: diffusion tensor imaging; fMRI: functional magnetic resonance imaging; CBT: cognitive behavioural therapy; RS-FC: resting-state functional connectivity; GAD: generalised anxiety disorder; PTSD: post-traumatic stress disorder; DZ: dizygotic; GSAD: generalized social anxiety disorder

Author	Year	Title of study	Main outcomes	Limitations of the study
Budisavljevic et al. [33]	2016	Heritability of the limbic networks	Inheritance of specific traits within the limbic networks is higher for the fronto-temporal pathways, which are responsible for complex social behaviour and emotional processing	Technical limitations of DTI tractography, Equal environment assumption, Twin studies applicable to non-twins, Only valid for the adult population, Limited by sample size, Predominantly female twins
Straube et al. [34]	2014	The functional – 1019C/G HTR1A polymorphism and mechanism of fear	GG carriers fMRI suggest increased activation of the amygdala before and after CBT. GG carriers also showed reduced effects from CBT	Small size of genotype subgroups, Activation of the parietal lobe can also be observed in healthy subjects and may be unrelated to CBT, Random allocation not stated
Trzaskowski et al. [35]	2012	Stable genetic influence on anxiety-related behaviours across middle childhood	Genetic factors (68%) were largely responsible for homotypic anxiety. Environmental factors were mainly responsible for heterotypic anxiety	Equal environment assumption, Chorionicity, Data collected using parent-rated questionnaire, Narrow period of investigation (7-9 years), Parental rating of similarity used (>98% of data contributed by mothers)
Sevi Tok et al. [36]	2016	The effectiveness of cognitive behavioural therapy, medication, or combined treatment for childhood anxiety disorders	All treatment methods involving CBT were more effective than standard drug treatment methods	Small sample size, Lack of placebo control condition, Based on self-report scales, Research data not collected by independent evaluators, Aged between 8 and 12 years
Stevens et al. [37]	2014	PACAP receptor gene polymorphism impacts fear responses in the amygdala and hippocampus	PACAP receptors modulate medial temporal lobe function in humans. Differences in ADCYAP1R1 contribute to abnormal fear circuitry playing a role in anxiety disorders	PACAP may influence estrogen levels/cycling, Representative of individuals who already had a significant level of trauma, Only African American women, Monetary compensation in an economically disadvantaged location
Burghy et al. [38]	2016	Experience-driven differences in childhood cortisol predict affect-relevant brain function and coping in adolescent monozygotic twins	Twin with higher childhood cortisol had lower rs-FC and poor amygdala recovery into adolescence following exposure to unpleasant images. Experience-dependent changes in the amygdala do not resolve into adolescence.	Small sample size, Focus on task-derived behaviour rather than psychopathology, Focus on experience – particularly early experience, Predominantly white subjects, Monetary compensation
Lewis et al. [39]	2014	Heritable influences on amygdala and orbitofrontal cortex contribute to genetic variation in core dimensions of personality	Genetic and environmental correlations between left medial orbitofrontal cortex thickness and negative emotions were observed. Heritable bases of personality are, in part, mediated through individual differences in the size of brain structures	Only representative of a small proportion of genetic and phenotypic variance between personality and brain regions, Left hemisphere showed greater association with personality -> no laterality hypothesis, Male-male only twins, Self-report questionnaire
Hettema et al. [40]	2012	A pilot multimodal twin imaging study of generalised anxiety disorder	GAD and genetic factors correlate with fear-related limbic structures and their connection with the frontal cortex	Small sample size, Few discordant pairs, Lack of info on current GAD status, Current medication use by some subjects, Women only, Caucasian subjects born in Virginia
Kuhlman et al. [41]	2014	Predicting developmental changes in internalizing symptoms: Examining the interplay between parenting and neuroendocrine stress reactivity	Parenting behaviours in early development, such as maternal warmth, lowered internalizing symptoms into preadolescence when risk for anxiety disorder onset is highest	Not representative of causal relationships, Low prevalence of clinically significant symptoms of internalizing, Large variation in maternal warmth symptoms, Only maternal reports -> no paternal, Parenting self-reported by mothers, No data on pubertal status of sample, Monetary compensation
Meirsman et al. [42]	2016	GPR88 in A2AR neurons enhances anxiety-like behaviours	GPR88 expressed in A2AR neurons enhances anxiety-like behaviours in mice without affecting fear responses	Animal study
Andero et al. [43]	2013	Amygdala-dependent fear is regulated by Oprl1 in mice and humans with PTSD	An SNP in Oprl1 is associated with self-reported history of childhood trauma and PTSD symptoms. Oprl1 is associated with amygdala function, fear processing and PTSD symptoms	Animal study, Human subjects were predominantly African Americans female
Davies et al. [44]	2015	Generalised anxiety disorder – a twin study of genetic architecture, genome-wide association and differential gene expression	A heritability analysis confirmed a significant genetic component contributing to anxiety sensitivity	Mostly DZ twins, Self-report questionnaires
Mansson et al. [45]	2016	Neuroplasticity in response to cognitive behavioural therapy for social anxiety disorder	Greater amygdala response in anxious patients compared to controls, before but not after CBT	Small sample size, Neuroplasticity and functional changes could be independently controlled by other processes, Eight individuals on psychotropic medication, Self-report questionnaires, Predominantly female subjects
Gingnell et al. [46]	2016	Combining escitalopram and cognitive behavioural therapy for social anxiety disorder: randomised controlled fMRI trial	Escitalopram and CBT results in more clinical responders, reduction in post-treatment anxiety and symptom severity. Therefore, adding escitalopram improved the outcome of CBT	Unable to directly compare combined with monotherapies, Did not include control for placebo+ICBT, Small sample size, Reported without corrected P-values could lead to false positives, Self-report questionnaires
Gorka et al. [47]	2015	Oxytocin modulation of amygdala functional connectivity to fearful faces in generalized social anxiety disorder	In individuals with GSAD, OXT enhanced functional connectivity between the amygdala and the bilateral insula during the processing of fearful faces	Small sample size, Male only subjects
Klumpp et al. [48]	2013	Neural predictors and mechanisms of cognitive behavioural therapy on threat processing in social anxiety disorder	Reduced activity following CBT. Pre-treatment cortical hyperactivity may be a prognostic indicator of CBT success	Lacked a wait-list control, Small sample size, 2 participants with GSAD on medication, No symptom improvement could suggest that neural changes directly correlate with treatment response

The role of limbic circuits and the HPA axis

Imaging using fMRI has helped study and understand the areas of the brain that are involved in the fear response and the responsiveness of these components in anxiety disorders [20]. The HPA axis plays a significant role in the generation of anxiety symptoms and its response [4]. Studies of limbic circuits in laboratory animals show a correlation with the brain activity of patients suffering with GAD, suggesting that limbic circuits play an important role in the fear response [49].

Budisavljevic et al. aimed to prove that the limbic pathways are heritable using an fMRI analysis of 26 monozygotic (MZ) and 17 dizygotic (DZ) twin pairs. Despite the conclusion stating that strong genetic effects were found to contribute to the anatomy of the limbic pathways, the data suggested that the only area of the limbic system to be predominantly affected by genetics was the uncinate fasciculus (UF) and genetic influence differed between the left and right side of the structures. Familial effects, such as shared-environment, were the predominant factor affecting the limbic system in this study [33]. Although this study only identified the UF as being genetically affected, cortical and subcortical structures are known to have significant heritability [50]. Along with the limitations, the study by Wen et al. opposes the results by Budisavljevic et al. and could discredit the accuracy of these results. Another important component of the limbic system is the HPA axis; stressful life events cause changes in the HPA axis contributing to the onset of anxiety disorders [51].

Kuhlman et al. hypothesized that environmental factors, such as parenting style, lead to a negative impact on the HPA axis. Results showed that increased maternal warmth led to decreased internalizing and anxiety-like symptoms, whereas an increase in internalizing symptoms caused a greater HPA-axis reactivity [41]. Cortisol levels were investigated in this study; symptoms of anxiety are associated with an increase in cortisol, and the early-life environment is the main determinant which controls the levels of cortisol exposed to individuals [52,53]. Kuhlman et al. showed that greater maternal warmth led to lower cortisol peaks into adolescence, although the literature and results supported the hypothesis, there were also several limitations to be considered. Perhaps one of the most important limitations of this study could be the self-reporting by mothers to determine parenting styles and anxiety behaviours; it is likely that parents would over-report socially desirable parenting, such as warmth, whilst under-reporting others such as physical punishment. This could be crucial in affecting the reliability of this study [41].

The hippocampus is another key component of the limbic system and HPA axis. Structural or volume changes in the hippocampus, as well as the size of the left and right hippocampi relatively, are a risk factor for the development of anxiety disorders [54]. Hettema et al. predicted that genetics underpins changes in the hippocampus in individuals suffering from an anxiety disorder. This twin study showed that the individual suffering from an anxiety disorder had smaller whole brain volume and smaller left hippocampal volume, thus increasing the risk of the development of anxiety disorders. Whilst the results support the hypothesis proposed in the study, it used 17 female-only MZ twin pairs [40]. This limitation could discredit the study as, on average, females have a smaller hippocampal volume, which could explain the results in the females suffering with anxiety disorders [55].

The amygdala and its connectivity

Straube et al. assumed that individuals with the HTR1A risk genotype (GG), which has been previously studied and shown to be a risk factor for anxiety disorders, would improve escape behaviour and reduce the effects of CBT. Results showed that carriers of the GG genotype escaped more than others, thus supporting their hypothesis [34]. In studies investigating the response of the amygdala in individuals with anxiety disorders, it was shown that amygdala response correlates positively with the severity of symptoms in GAD [56]. Another study investigating the activation of the amygdala in a mixed cohort of individuals suffering with GAD and SP, also with a low tolerance for uncertainty, showed that during a decision-making task, there was increased amygdala activation [57]. Supporting this literature, Straube et al. showed that GG carriers had greater activation of brain regions; however, the activation of the amygdala remained constant following CBT, implying that CBT was less successful for these individuals [34]. On the other hand, CBT has been demonstrated as strengthening the connections between the amygdala and other brain regions, which could explain the prolonged activation [58]. Similarly, the results of a study performed by Mansson et al supported the physical, structural and connectivity changes to the amygdala caused by CBT [45].

Lewis et al. performed a male-male twin study aimed to determine the genetic influence on positive and negative emotionality. The study showed a positive correlation between genetics and positive or negative emotionality. Significant correlations between the environment were also noted. The study also identified increased right amygdala activation with positive emotionality. Whilst the fact that this is a male-only study is a significant limitation and makes it difficult for these results to represent the whole population, it allows for the substantiation of findings in other studies [39]. No significant relationship has been shown between the right amygdala and emotionality in women, but studies have shown this relationship in men [59]. 

The amygdala functions by connecting with the brain regions responsible for the interpretation of social behaviour [16]. Studies of the amygdala in humans have implicated this mechanism in the recognition, expression and experience of fear, similar to midbrain and brainstem structures. The amygdala’s role in fear response is comparable across species from humans, to rodents and even reptiles, mirroring the pattern of connectivity [18,60]. Reduced connectivity between the amygdala and its brain regions has been implicated as a risk factor for the onset of anxiety disorders [61].

Stevens et al. and Burghy et al. implicated the amygdala and its connectivity with brain regions in their investigations [37,38]. Stevens et al. believed that individuals with the risk polymorphism would show reduced functional connectivity between the amygdala, hippocampus and medial prefrontal cortex. The amygdala to hippocampal connectivity was reduced in the risk group; however, no amygdala to prefrontal cortex connectivity was influenced by the risk group [37]. In contrast to this, Burghy et al. identified that environmental factors, such as early childhood experiences, impact the connectivity between the amygdala and the prefrontal cortex, suggesting that prefrontal connectivity is significantly influenced by environmental factors rather than genetics [38]. Early childhood experience is thought to cause mature connectivity between the amygdala and other brain regions [62].

Some treatment therapies are thought to work by changing amygdala connectivity to aid anxiety symptoms. Gorka et al. hypothesized that oxytocin (OXT) would affect amygdala function and their results supported this as those consuming OXT had increased functional connectivity compared to healthy controls. This study was limited by the subject group being male only [47]. Klumpp et al. implied that treatment with CBT would affect connectivity and that changes in brain regions would correlate with symptom severity. CBT success was indicated by the hyper-reactivity in the amygdala. Individuals reported reduced symptom severity; although brain activity did not correlate with this, this was predicted to be due to varying individual perceptions of fear [48].

Specific genes and anxiety disorders

Twin studies have helped distinguish the genetic from the environmental contribution in the transmission of anxiety disorders to offspring. Focusing specifically on the risk of offspring, those who have parents suffering with at least one anxiety disorder have a greater risk of also developing an anxiety disorder [10]. Homotypic continuity of anxiety disorders is the continuation of an anxiety disorder over time, whilst heterotypic continuity is the development of one anxiety disorder to another. Trzaskowski et al. predicted that homotypic continuity of anxiety disorders is largely due to genetics, whereas heterotypic continuity is less so. Results show that genetics contribute to around 57% of homotypic continuity, whilst heterotypic continuity was largely caused by environmental factors. Although there seems to be no recent conflicting literature, this twin study is based on the equal environment assumption, implying that the twins involved in the study are under the exact same environmental influences [35].

Meirsman et al., Andero et al. and Davies et al. identified specific genes in the regulation, development and risk of onset of anxiety disorders [42-44]. Meirsman et al. found that Gpr88 expression in neurons is responsible for anxiety-like behaviours in a subject cohort of male mice. This is a significant limitation of this study and further research on human subjects may be required [42]. Andero et al. assumed that Oprl1 is associated with the connectivity of the amygdala, leading to the onset of anxiety disorders. Whilst the polymorphism in this gene showed greater functional connectivity between the amygdala and its receptor, trauma prior to the onset of clinical anxiety begins to alter the expression of Oprl1 [43]. No limitations were noted in the study, and a study by Hamm et al supported the results of this study, as hyperconnectivity in the amygdala in children prior to anxiety disorders was recognised as a risk factor for its onset [63].

Davies et al. performed a twin study focused on gene expression and associations with anxiety sensitivity in MZ twins. Polymorphisms within the RBFOX1 gene impact the expression of this gene within the limbic system, affecting neuronal brain activity. Investigation into anxiety sensitivity has shown that although both twins have polymorphism in the RBFOX1 gene, they are expressed differently and only one twin has clinically significant symptoms of an anxiety disorder. The results of this study seem to contradict one another; whilst the RBFOX1 gene was shown to increase the risk of anxiety in individuals, it does not always cause expression of the anxiety phenotype [44]. However, it is common for MZ twins to express different phenotypes to the same gene or its polymorphisms, perhaps explaining the results found by Davies et al. [64].

Substantial progress has been made in the majority of areas of psychiatric genetics; however, very few genetic causes have been identified in anxiety disorders. This is likely to present challenges in future research due to the complex overlap between anxiety disorders and the significant environmental contribution [65].

Treatment of anxiety disorders

Anxiety disorders can be treated with psychological, pharmacological methods or a combination of the two. Cognitive behavioural therapy (CBT) is usually the first-line choice for psychological treatment, whilst selective serotonin reuptake inhibitors (SSRIs) are the first-line choice for pharmacological treatment [66]. Pharmaceutical and psychological therapies are independent of one another, and studies have found that neither is more effective than the other; however, combination therapies have been shown to be twice as effective as placebo only [67].

Sevi Tok et al. and Gingnell et al. considered the effect of combination therapies compared to single therapies in the effectiveness of treating anxiety disorders [36,46]. Sevi Tok et al. hypothesized that a combination of talking therapy and standard pharmaceutical therapy would be more effective in treating the symptoms of anxiety and improving the quality of life. The results show that combination therapy decreased anxiety-like symptom severity compared to single therapy groups and this continued into the monitoring phase [36]. Gingnell et al. hypothesized that adding escitalopram to CBT would enhance its anxiolytic effect and lead to a decrease in amygdala activity. The combined therapy led to increased response to CBT by participants and reduced amygdala activity [46]. However, both of these studies had similar limitations of small sample size and lack of placebo control, leading to the question as to whether these studies could be representative of the whole population and the reliability of the results [36,46].

Challenges for healthcare professionals in treating anxiety disorders include inappropriate diagnosis and treatment of the disorders, poor engagement with psychological and pharmacological and limited evidence to support the effectiveness of treatment [68]. More recently, Levey et al. have performed large-scale genomic studies that will aid in the development of therapeutic pathways [69]. Considering all of these challenges, it is important to allow patients to make informed decisions on their management plan [3].

Strengths and limitations

This research benefitted from the large amount of research which has been done into anxiety disorders and its effects on individuals. A significant amount of research was found to implicate the importance of the amygdala and its connectivity within fear circuitry, as well as the importance of the brain regions in memory formation. Treatment methods have also been vastly studied, in particular, talking therapies, such as CBT, allowing these treatment methods to be associated with environmental factors.

Despite the extensive amount of research to help support this project, there were also significant weaknesses. Twin studies were ideal to determine the causal factors of anxiety disorders; however, the number of recent twin studies available relating to anxiety disorders and fear circuitry was limited. Of the twin studies that were used, sample size was generally small and gender-specific. A significant number of the genetic studies used animal subjects; although brain regions are thought to be near identical across mammalian species, it is difficult to reliably use results from animal studies to represent the general human population.

## Conclusions

Further research will benefit from determining the exact process which causes the development of anxiety disorders to facilitate detection and intervention before it results in life-long impacts on patients and has negative consequences on future generations. Larger sample sizes and more varied subject participants would be advantageous.

To conclude, the above research seems to show no clear answer to the question at hand. However, a significant amount of research has implicated a combination of both genetics and environmental factors in the onset and development of anxiety disorders. The research suggests that whilst a genetic predisposition for anxiety disorders is indisputable, environmental factors, such as childhood experiences or adverse events, could then lead to the presentation of an anxiety disorder.
